# Balint style case-based discussion group for medical students in Bassetlaw Hospital

**DOI:** 10.1192/bjo.2021.898

**Published:** 2021-06-18

**Authors:** Anu Priya, Hardev Bhogal

**Affiliations:** Bassetlaw Mental Health Services

## Abstract

**Aims:**

To evaluate feedback from Balint style case based discussion groups and to reflect on learning points.

We have three medical students from Sheffield University on six week psychiatry placements at Bassetlaw Hospital and we get eighteen students in a year. In order to further develop their approach towards reflection and their understanding towards the doctor patient relationship we developed the Balint style case based discussion group, and each group of students attend three sessions during their placement.

**Method:**

The groups are held on a weekly basis and consist of the three medical students and 1-2 facilitators. As the group is small one of the facilitators may participate with the students for the Balint process and to help encourage the students. Following completion of the third session of the discussion group we gain written feedback from the students. A total of 17 feedbacks have been reviewed over the period of November 2018 -November 2019.

**Result:**

16 students stated that this was their first experience at Balint Group and all except one student felt that they were given a good introduction about Balint groups before starting. When asked about the most significant thing that they have learnt in the group, the majority of students marked reflecting feelings to improve relationships with patients, exploring why they feel a certain way with patients and that the doctor patient relationship can affect the consultation.

One student stated that they would not recommend it to colleagues as they felt it was relevant more to doctors rather than medical students. Another student recommended having more people in a group.

**Conclusion:**

Overall, it has been a positive experience with the medical students during the groups and with feedbacks. We have reflected on difficult topics like bereavement, fantasized about the purpose of a patient's delusion and shared the joy of a patient who was discharged after a long stay. While we think we have been able to teach the students some tips on reflection, we ourselves have been able to reflect on certain topics we would not have if not raised by the students. Some medical students have contacted the larger Balint Group in Sheffield for further sessions. Considering our experience, we will continue with the sessions at Bassetlaw Hospital.

